# Chlorpyrifos and neurodevelopmental effects: a literature review and expert elicitation on research and policy

**DOI:** 10.1186/1476-069X-11-S1-S5

**Published:** 2012-06-28

**Authors:** Margaret Saunders, Brooke L Magnanti, Sara Correia Carreira, Aileen Yang, Urinda Alamo-Hernández, Horacio Riojas-Rodriguez, Gemma Calamandrei, Janna G Koppe, Martin Krayer von Krauss, Hans Keune, Alena Bartonova

**Affiliations:** 1 University Hospitals Bristol NHS Foundation Trust, England; 2 NILU - Norwegian Institute for Air Research, Kjeller, Norway; 3 Instituto Nacional de Salud Pública, Mexico; 4 Instituto Superiore di Sanità, Rome, Italy; 5 Ecobaby Foundation, Netherlands; 6 World Health Organisation, Copenhagen, Denmark; 7 Research Institute for Nature and Forest (INBO), Brussels; Centre of Expertise for Environment and Health, Faculty of Political and Social Sciences, University of Antwerp; naXys, Namur Center for Complex Systems, University of Namur, Belgium

## Abstract

**Background:**

Organophosphate pesticides are widely used on food crops grown in the EU. While they have been banned from indoor use in the US for a decade due to adverse health effects, they are still the most prevalent pesticides in the EU, with Chlorpyrifos (CPF) being the most commonly applied. It has been suggested CPF affects neurodevelopment even at levels below toxicity guidelines. Younger individuals may be more susceptible than adults due to biological factors and exposure settings.

**Methods:**

A literature review was undertaken to assess the evidence for CPF contributing to neurodevelopmental disorders in infants and children. Other literature was consulted in order to formulate a causal chain diagram showing the origins, uptake, and neurological effects of animal and human exposure to CPF.

The causal chain diagram and a questionnaire were distributed online to scientific experts who had published in relevant areas of research. They were asked to assess their confidence levels on whether CPF does in fact contribute to adverse neurodevelopment outcomes and rate their confidence in the scientific evidence. A second questionnaire queried experts as to which kind of policy action they consider justifiable based on current knowledge. In a special workshop session at the EuroTox congress in Dresden in 2009 the results of both questionnaires were further discussed with invited experts, as a basis for a policy brief with main messages for policy makers and stakeholders.

**Results:**

Most experts who responded to the first questionnaire felt that there was already enough evidence to support a ban on indoor uses of CPF in the EU. However, most felt additional research is still required in several areas. The responses from the first questionnaire were used to formulate the second questionnaire addressing the feasibility of government action. In turn, these expert participants were invited to attend a special session at the EuroTox congress in Dresden in 2009.

**Conclusions:**

Some of the evidence that CPF contributes to neurodevelopmental disorders is still disputed among experts, and the overall sense is that further research and public awareness are warranted. There have been campaigns in North America making the potential exposure concerns known, but such information is not widely known in the EU. The ability of government action to produce change is strongly felt in some quarters while others believe better knowledge of consumer use trends would have a greater impact.

## Background

### Introduction

Organophosphate (OP) compounds are used worldwide in agriculture and gardening to control insect pests. They also have residential and indoor applications for pest control, especially for cockroaches and termites [1-6]. OPs act by inhibiting acetylcholinesterase, thus affecting nerve function in insects, humans and other animals. Most of the animal and human studies published from 2000 onwards refer to the OP chlorpyrifos (CPF).

There is concern about the safety of CPF in the environment. While previous studies have shown levels of CPF that are safe in adult animals, recent evidence indicates young animals and humans may be more sensitive to CPF toxicity. In young animals, CPF is neurotoxic and mechanistically interferes with cellular replication and differentiation. This leads to alterations in the synaptic transmission in neurons.

OPs are used frequently in Europe for pest control due to their low price and broad spectrum of activity. In 2003 they accounted for over 59% (4645 tonnes) of insecticide sales in the EU, with CPF the top selling insecticide (15.6%, 1226 tonnes) [7]. CPF was also one of the most widely used OPs in the US for pest control [2], but the US Environmental Protection Agency (EPA) imposed a ban on the sale of CPF for residential use in December 2001 [8].

The consideration of whether to ban OPs for domestic use in Europe is a complex process involving both health and lifestyle considerations. Moving from scientific data to policy interpretation is a nontrivial challenge, because public health risks are scientifically very complex. Scientific assessment of environmental health risks is faced with large, sometimes irreducible, uncertainties, knowledge gaps, and imperfect understanding, and may also have conflicting claims and scientific controversy [9,10].

The HENVINET project focussed on the four priority health diseases of the European Environment and Health Action Plan (EHAP) 2004-2010. These are: asthma and allergies, cancer, endocrine disrupting effects, and neurodevelopmental disorders. Because CPF is one of the key environmental pollutants strongly connected in the scientific literature with neurodevelopmental effects, and because of the North American ban on its domestic use, HENVINET chose to focus on this pesticide in particular.

In order to better inform policymakers of the scientific basis of any proposed action, an expert elicitation was undertaken to identify areas of the research in need of further examination. This study considers the environmental health effects of CPF exposure in utero and during childhood and its relationship with neurodevelopment. The results will be used to form the basis of a decision support tool that has the aim of preparing policymakers with the necessary scientific background to address the concerns surrounding OPs and their applications in the home.

### Scientific Background

Activities involved in the production, storage, transport and use of CPF may play a role in its release as it is transferred from the production site to the final user. Unintentional release through dumping or leakage can lead to unexpected exposures. The uptake of CPF into the environment depends on factors such as the strength at the source and the physical form (dry solid, liquid, etc.). The extent of use will also depend on the time and location. For example, agricultural and gardening use will be influenced by the seasonal growth of crops and plants, whereas residential use is less likely to be specifically influenced by the seasons apart from the climate effects on pest infestation. There may still be a seasonal influence on child exposure.

### Environmental matrix

Dispersion and transformation of CPF from the sources affects uptake into the environment and may be influenced by transport, climate and the characteristics of the area where they are being applied. The use of CPF for agricultural and gardening purposes will lead to accumulation in soil, water and on food such as vegetables and fruit as well as atmospheric dispersal [1-5].

However, residential use is considered to be the main source of contamination in the majority of the population, alongside contaminated food consumption [5]. This can lead to accumulation in indoor air, including house dust, and on surfaces including toys [2,4].

Incorporation of CPF into each environmental matrix will vary according to concentration and is influenced by composition (parent compound/environmental metabolite), how the load is spread (concentrated or dispersed), and the magnitude of the load and the frequency of application.

### Exposure setting

Population behaviour influences interaction between the environment/exposure setting and the extent of exposure. For CPF, there are three key exposure settings: occupational, ambient and indoor.

Occupation puts farming and greenhouse workers at risk from sources used in agriculture and gardening. Similarly, manufacturing workers are also at risk of exposure, especially if there is an inadvertent leak. The general public, especially children, are mainly at risk from ambient and indoor residential exposure. Several physical processes related to the types and settings of these exposures are also possible.

Oral exposure can arise particularly from fruit and vegetables consumed as part of the normal diet, but also water, milk and derived products [2,4]. Indirect exposure occurs within the ambient and indoor settings [2-5]. Non-dietary oral exposure (contact with soil and household objects) is an important exposure route for younger children due to their behaviour patterns with respect to play at floor level and on/with other surfaces and toys. Inhalation of indoor air is another route with house dust a critical component. Dermal exposure is also possible through this route [11].

A 1993 review conducted by the US Commission of Life Sciences examined organophosphate pesticide exposure routes in infants and children. For six pesticides (chlordane, heptachlor, aldrin, chlorpyrifos, diazinon, and gamma-BHC), the mean air exposures were consistently higher than the estimated dietary exposure for the same chemicals [12].

One study measured chlorpyrifos concentration following flea treatment in a carpeted home. They found CPF vapours in the infant breathing zone (25 cm above the carpet) significantly higher than those measured in the adult breathing zone. Time-weighted averages for the 24 hours following application in the infant breathing zone were 41.2 and 66.8 µg/m3 for ventilated and nonventilated rooms, respectively. This is far higher than the guideline of 10 µg/m3 proposed by the U.S. National Research Council's Committee on Toxicology [13]. Also, air concentrations increased up to 5 to 7 hours after application. This suggested treated carpets are a source of volatilised chlorpyrifos and even with open windows, concentrations nearest the floor remain high [14].

In assessing risk for infants in chlorpyrifos-treated homes, Berteau et al. [15] calculated an absorbed dose of 2.68 mg/kg. Fenske et al. [14] found the estimated absorbed chlorpyrifos dose for infants exceeded the EPA's no-observed-effect level of 0.03 mg/kg/day in each case. The level is based on measured changes in plasma acetylcholinesterase.

The indoor use of pesticides in public buildings is another source of exposure. Employees of one school became ill within hours of entering a building that had been treated for roaches 3 days earlier and had not been ventilated. It was 14 days before air levels of the pesticides decreased to an acceptably safe level and students were readmitted [16]. An air analysis indicated that the levels decreased at a much slower rate than indicated by the manufacturer’s guidelines.

Indoor insecticide sprays and foggers persist on carpets, floors, and other surfaces in the home. Young children wearing only diapers may experience dermal exposure playing on previously sprayed surfaces; children who put their mouths on objects may ingest the substances. In one case, pesticide poisoning was suspected when an infant suffered respiratory arrest and tests showed his red blood cell cholinesterase levels depressed to 50% of normal levels. The child's home had been treated with chlorpyrifos and the chemical was subsequently found on dish towels, food preparation surfaces, and the infant's clothing [17]. Flea control products persist on a pet's fur and could be transferred to children [18].

Exposure during pregnancy is an area of concern given the high percentage of women using pest control during pregnancy and the vulnerability of the foetus during development. Foetal exposure occurs through transplacental transfer with the placenta failing to act as a barrier to lipophilic OPs [6]. There is limited data concerning the presence of OP in human breast milk [19], possibly due to partitioning into the water fraction of breast milk. This area requires further investigation as it may present an additional exposure route during the postnatal period [20].

The extent of exposure will be affected by the frequency, duration and intensity of contact, which can all vary. There may also be transfer between settings. For example, a parent who is an agricultural worker may transfer residue to their offspring within the home.

Geographical location of the setting may also play a role as the dissipation of CPF and its metabolites from food surfaces has shown a wide range of variation, with shorter times shown in more tropical climates [21], and longer times in more temperate ones as well as on foods cultivated in winter [22].

### Toxicokinetics

The dose of pesticides in organs and tissues is determined by the pharmacokinetics of CPF: physical absorption, distribution, metabolism and excretion processes following uptake. An important element in assessing exposure is the biological matrix used for sampling. Levels in humans are determined through biomarkers which may be subject to interpretation.

For CPF, the most commonly used biomarkers are found in blood and urine. In blood, exposure is determined by measurement of plasma butylcholinesterase (BuChe) activity and erythrocyte acetylcholinesterase (AChE) activity [23]. Urine measurements detect excretion of metabolites and are more widely used for young children compared with taking blood samples. CPF is activated in the liver to CPF-oxon by cytochrome P450-dependent desulfuration [24].

The most sensitive biomarker for testing foetal exposure is meconium [6,25] as compared with the sensitivity found in testing cord blood [26]. When tests of meconium are combined with other markers such as maternal hair samples, the detection rate is further increased [27].

Measurements of CPF or CPF-oxon are the most specific marker for exposure [28]. However, organophosphates are rapidly metabolized in the body and almost entirely excreted in the urine [3]. Some may be stored in adipose tissue [28], meaning that parent compound levels in blood are very low compared with metabolites.

The CPF metabolite 3-5-6 trichloro-2-pyridinol (TCPy) can be detected in urine [29,30] as can the non-specific OP dialkyl phosphate (DAP) metabolites formed from nearly all OP insecticides [5]. For CPF, these DAP metabolites are diethylphosphate (DEP) and diethylthiophosphate (DETP). However, about 75% of OP pesticides are also biotransformed to DETP, DEP or other DAPs measured in the same way and they cannot be distinguished from environmental degradates [24]. Careful interpretation is needed when measuring DAPs as they cannot necessarily be correlated with specific OP insecticides and the metabolites themselves may be ingested [5].

Route of exposure affects absorption and hence body burden and target organ dose. A case study of CPF and malathion biomonitoring demonstrated that about 70-93% of the oral dose of CPF could be recovered in the urine compared to only 1-3% of the dermal dose [28]. Pharmacokinetics also influences organ dose and effective dose through distribution, metabolite production and enzyme function. OP pesticides can be converted to oxon form which interacts with cholinesterase. However, the oxon form can also be enzymatically or spontaneously hydrolysed to form a DAP metabolite and an organic metabolite. Unconverted OP can also be hydrolysed to the organic group metabolite and DAP metabolites [28]. These metabolites or their conjugates are excreted in urine. There will also be differences between foetus, newborn, child and adult metabolisms and how such metabolites are cleared by kidneys from the system.

### Health effects

Age and genetic/acquired predisposition may determine health effects from the CPF exposure dose. CPF toxicitiy is due to the inhibition of acetylcholinesterase by the CPF-oxon, preventing efficient degradation of acetylcholine and leading to accumulation of transmitter molecules in the nerve synapse. Elevated synaptic acetylcholine levels result in persistent receptor stimulation and the alteration of signalling pathways with functional changes at tissue/organism level [29].

While some researchers have asserted that CPF does not affect any biological systems at levels below those established for extreme cholinesterase inhibition and acute toxicity [30], numerous animal and in vitro studies suggest that CPF can act by other mechanisms. They have also demonstrated that CPF exposure at doses below the threshold for systemic toxicity and inhibition of brain cholinesterase exerts disruptive effects on neural cell development, with respect to DNA synthesis, gene transcription, cell differentiation, and synaptogenesis [31]. These effects are particularly enhanced in early development.

Several rat studies have indicated that CPF targets neurotransmitter systems further to the cholinergic one, as the monoamines, norepinephrine, dopamine, and serotonin [32]. In addition, glial cells are more sensitive to CPF than neurons and may be preferentially targeted [33]. Interference with brain maturation is associated with behavioural disturbances in exposed rodents, including hyperactivity, learning impairment and alterations in the social and emotional domain [34-39]. This suggests vulnerability during foetal and childhood periods [40]. CPF is considered moderately toxic and is an EPA class II toxicant i.e. oral dose LD50 is 50-500mg/kg [28].

### Juvenile and prenatal susceptibility

Animal studies have demonstrated that juveniles are more susceptible to OP toxicity than adults [41]. Animal and in vitro studies show low-dose OP exposure in developmental periods produces neurochemical and neurobehavioural changes [40], even at doses below what would ordinarily produce detectable changes in brain acetylcholinesterase (AChE) [39,42]. Changes such as the morphology of the hippocampus and levels of neural growth factor [43], excess weight gain [44], and changes in anxiety, maternal behaviour and social responses [45] have been observed. These suggest interference with hypothalamic neuroendocrine mechanisms. Differences in young animals are attributed to incomplete metabolic competence during development [46] and the susceptibility of the rapidly developing nervous system.

Paraoxonase 1/arylesterase (PON1) is a key OP detoxifying enzyme. Increased sensitivity to OP toxicity in newborns may be due to reduced PON1 levels, which are 3- to 4-fold lower than in adults. There is considerable PON1 polymorphism and this genetic variability will affect sensitivity alongside a 13-fold variation in adult levels [41,47].

Additional noncholinergic mechanisms - such as oxidative stress - may damage the developing brain with exposures occurring below the systemic effects threshold. Thus nonsymptomatic exposure for pregnant women, infants and children and could be linked with increased risk for development of metabolic diseases such as diabetes [48].

Pre- and postnatal exposure has been linked with developmental disorders in children. Prenatal residential exposure to CPF of inner city children assessed at age 3 years was linked with impaired motor skills and impaired mental development. Highly exposed children are more likely to exhibit clinical symptoms of attention problems, ADHD and pervasive developmental disorders [20].

In utero exposure of children born in an area of major agricultural production was associated with impaired reflex functioning, particularly in those assessed after 3 days postnatal [49]. Organophosphate poisoning in children under the age of 3 was linked with impaired verbal learning and motor inhibition tasks, with higher impulsivity in OP intoxicated children [50]. Pervasive developmental disorders at age 24 months were found to be linked with uterine exposure in Mexican-American children [51].

In mother-infant pairs exposed to indoor residential pesticide exposure, a positive trend was found between maternal PON1 activity and head circumference in offspring where maternal CPF metabolite (TCPy) were above the limit of detection [40]. Eskenazi et al (2004) [52] found an association between increased levels of dimethyl phosphate metabolites (coming from malathion) in the urine in later pregnancy and a reduced gestational duration.

Also in that study a reduced length of gestation was found in relation with the cholinesterase levels (ChE) in umbilical cord whole blood. Maternal dialkyl phosphate metabolite levels and ChE levels in later pregnancy were not correlated. Unexpectedly, there was a positive effect of the dialkyl phosphate metabolite levels on head circumference after correction for creatinine levels. In contrast, Whyatt et al in 2005 [53] found a significant inverse correlation between cord blood plasma CPF levels and birth weight and length for children born before the 2001 ban. Later follow-up of this group revealed neurodevelopmental abnormalities at the age of 3 in relation to prenatal exposure to CPF parent compound as could be expected considering the intra-uterine growth retardation [20].

## Methods

The objective of this phase of the project was to identify areas of knowledge gaps in order to prioritise issues for further attention from scientists and policymakers. The central focus developed for the HENVINET project was to question which kind of policy action experts consider to be justifiable based on the identified state of scientific knowledge; the societal impacts and aspects were not initially addressed in depth, with the focus mainly on knowledge gaps, and thus to science itself. One challenge of such a study is the state of knowledge about health risks caused by environmental pollution and contaminants, and how to tease out the specific contributory effects of CPF. The question of what to do with a large and complex amount of data remains difficult. Measuring pollutants and related health effects presents its own challenges, but how should scientists interpret these results, and how should decision-makers translate them into policy?

So within this phase of work, the scientific data were considered specifically, with the main aim to be identifying areas of controversy and need for further research. Further deliberation with other stakeholders would need to occur before specific policy recommendations could be put forward.

One influential model of expert elicitation is given in the RIVM Letter Report [54]. It details a process by which uncertainties related to the question or problem are considered, and the need to perform expert consultation identified. Then three actions are applied: the selection of experts, the identification of key uncertainties for discussion, and the assembly and dissemination of basic information.

For a more detailed explanation of HENVINET’s elicitation process and the social science issues involved please see the Keune, Gutleb et al. paper elsewhere in this volume, “We’re only in it for the knowledge? A problem solving turn in environment and health expert elicitation”.

### Selection of experts

Subject-matter experts to assess the scientific case were selected through identifying primary authors of published literature in the field, as well as key and well-known researchers in industry. A number of experts who were approached were already in the HENVINET consortium with a majority not involved. Most of those approached were located in North America and Europe with a few from Asia. About 40 experts were approached by an initial letter detailing the aims of the project and describing the basic information and a questionnaire, which were made available online.

### Dissemination of basic information

**Figure 1:**
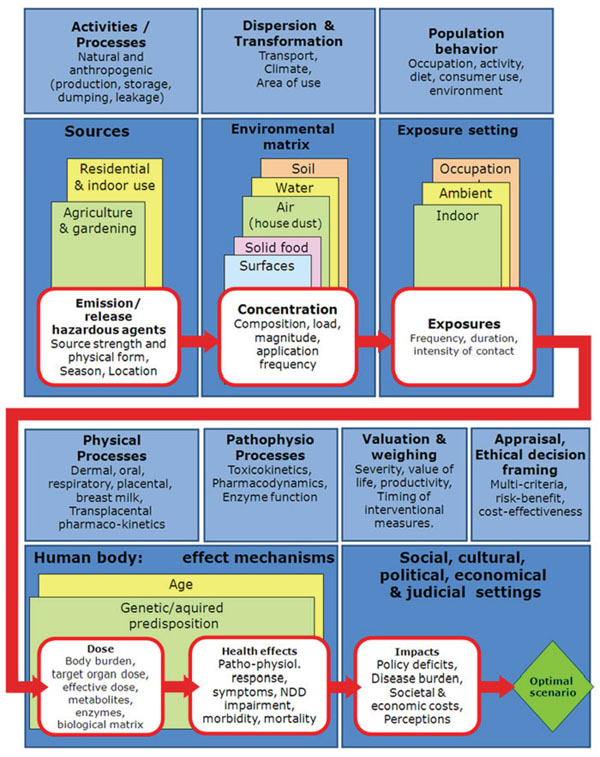
**Causal chain diagram for the insecticide Chlorpyrifos** This causal diagram addresses the agent chlorpyrifos (CPF) and the evaluation of the knowledge quality used to derive the causal chain.

A literature review of the evidence regarding the contribution of Chlorpyrifos (CPF) to developmental disorders was undertaken. Details of the content of the review are in the section Scientific Background above. Using this review, a causal chain diagram (Fig. 1) was formulated to be distributed to experts for their commentary and suggestions.

The route of dissemination was through the HENVINET web portal, where elements of the causal chain diagram and the diagram in its entirety were presented alongside the literature review. Elements of the diagram were assessed for completeness, accuracy, and availability of knowledge in the specified area of research.

### Identification of key uncertainties

The questionnaire asked experts for their opinion of the quality of available evidence for key areas of certainty about the quality of evidence in the causal chain to be assessed on a scale of five ranging from Very High certainty to Very Low certainty.

The results of the questionnaire were tabulated in order to identify focus areas of uncertainty and lack of knowledge for further discussion. Using a pie chart representation, it was easy to visualise the spread of opinions in the area. Questions in which there was nonconsensus regarding certainty of the quality of knowledge were characterised by having a spread of answers across all possible answers. Questions in which there were disagreements were characterised by answers clustering in the ‘Very High’ and ‘Very Low’ groups with little in between.

## Results

### First questionnaire

**Figure 2:**
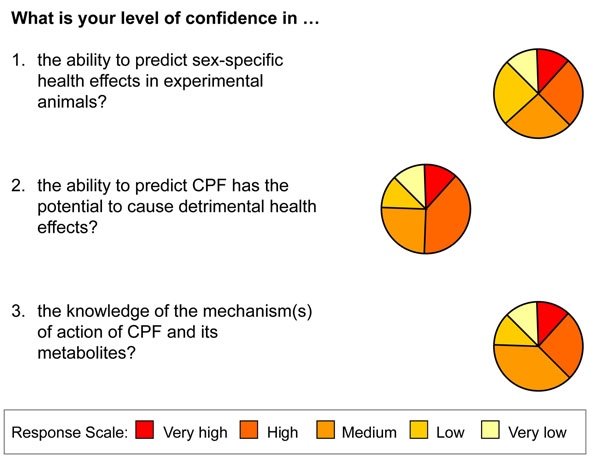
Questions regarding the level of confidence

Of interest were areas in which there was disagreement between experts, where the consensus regarding the reliability of the scientific evidence could not be achieved. Here are questions in which there was high disagreement (Fig. 2).

**Figure 3:**
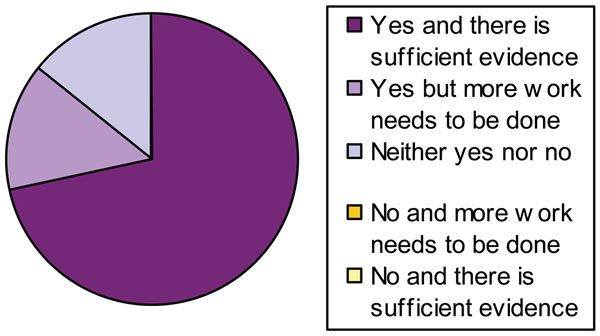
Level of agreement about whether CPF should be banned from home use

There were also areas of high agreement. Experts considered the quality of evidence for a clear risk, results of which varied from *very high* confidence to *very low*. Many felt more research was necessary to quantify the risk. However, when asked *whether CPF should be banned from home use*, the majority agreed (Fig. 3).

**Figure 4:**
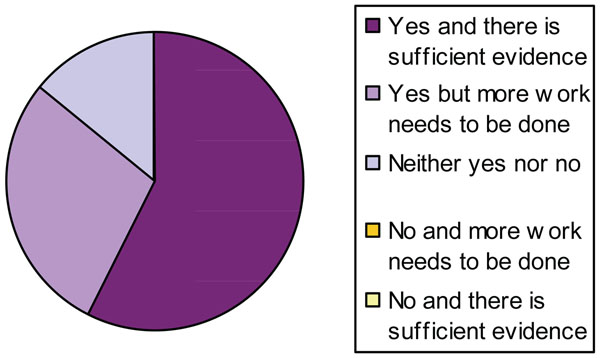
Level of agreement about if CPF should be banned due to specific neurodevelopmental effect

None of the experts chose the ‘No, and more work needs to be done’ or ‘No, and there is sufficient evidence’ options. When asked if CPF should be banned due to *specific neurodevelopmental effects*, again the majority agreed (Fig. 4).

### Second questionnaire and workshop

An expert consultation and second questionnaire on policy action followed the first questionnaire. Two respondents attended the workshop along with a social scientist and a consortium moderator. The participants represented the farthest ends of the continuum from the first questionnaire. The depth of examination in such a group can help to identify areas of concern where perhaps a larger group would not be able to explore such issues.

Experts agree that the three priority areas to investigate are:

• *Population behaviour*, including occupation, diet, and at-home use,

• *Physical processes*, such as uptake or absorption, since these determine exposure, and

• *Pathophysiological processes*, like enzyme function, which determine exposure outcome

When asked what has the greatest effect on health risks from CPF, these were identified:

• *Population behaviour*, including occupation, diet, and at-home use,

• *Physical processes*, such as uptake or absorption, since these determine exposure, and

• *Pathophysiological processes*, like enzyme function, which determine exposure outcome

Pre- and post-natal exposures were considered important. Specific questioning for more detail revealed:

• ‘Frequency and duration of exposure… affects health risks’

• ‘Age and genetic polymorphisms influence toxicity’

• ‘More research needed… in low doses of chlorpyrifos.’

More research was recommended regarding specific EU indoor exposures to CPF. It was also discussed whether CPF is the causal toxin or if it is a proxy in studies for some other exposure or behaviour. Merits of particular study designs were discussed.

It was felt both research and policy action can contribute to reducing problems. One scientist commented changes in policy were ‘feasible immediately’. More data about exposure, better scientific understanding, and CPF monitoring were supported.

Further comments included ‘I think CPF is fine for outdoor use… indoor use is of concern.’ Another suggested ‘strict evaluation of current use in… domestic settings.’

## Discussion

A number of recommendations resulted from the previous elicitation and the content of the conference session.

When it came to assessing the areas of most concern to experts working in the field, population behaviour and physical processes were considered the most important factors in toxicological outcome.

Several areas showed important differences in terms of expert opinion. The arguments against an indoor use ban in the EU included the opinion that there are limited data on effect at low, sub-toxic levels but also a request for more epidemiological evaluation of the risk issue. Other experts were confident that there are already enough data to go ahead with a restriction on use. But all agreed that because much of the epidemiological research has taken place in North America rather than Europe and the rest of the world, more focus and funding in the future should be addressed on design of studies being appropriate to realistic exposures in the home that are suitable to the EU.

Experts suggested more scientific research with focus on more data and better understanding of fundamental science – both in the cases of those opposed to and supporting a restriction on CPF use. There was also a request for policy action, especially more monitoring activities, but also the possibility of revisiting the issue on a regular basis in order to assess the need for some restricting and prohibiting activities.

Research to determine whether factors influencing use of CPF in North America are applicable as a form of action, to determine whether exposure at a sub-clinical level has a measurable effect. The use of policy to decrease or stop this exposure by raising awareness and restricting certain activities was supported even by sceptics of CPF’s effects on neurodevelopment.

The experts have some confidence in science coming up with usable or decisive knowledge within the next five years, provided research continues to be supported and addresses the focus outlined above for EU-specific, realistic-use epidemiology as well as laboratory studies.

## Conclusions

As indoor usage restrictions for CPF have been considered and rejected earlier in the EU, there are questions as to whether policy makers could be motivated to re-examine this topic as most participants responded that policy could have a significant impact. Policy makers must decide whether CPF’s negative effects are worth reconsidering and the possibility of a ‘silent epidemic’ is something they feel comfortable continuing to ignore.

## Competing interests

Authors declare that they do not have any competing interests.

## Authors' contributions

MS was responsible for study design, literature review, causal diagram development and manuscript writing. BLM was responsible for questionnaire design, data analyses, results interpretation and manuscript writing. SCC contributed to the design of the questionnaire and the causal chain diagram, as well as to expert recruitment. AY contributed to the causal chain diagram and questionnaire development and implementation, and to first questionnaire analysis. UAH and HRR contributed to literature review, causal diagram development and questionnaire for expert revision. GC contributed to literature review, causal diagram development, preparation of the first questionnaire for experts and manuscript revision. JGK contributed to the literature review, causal diagram development and second workshop, and manuscript revision. MKK contributed with questionnaire development, more generally on methodology and manuscript revision. HK was responsible for development of the second questionnaire, the workshop and methodological evaluation of workpackage 1. Other coauthors participated in the study design, data collection and results interpretation. AB contributed with concept development, overall structure of the study, data interpretation and was the project coordinator.

All authors read and approved the final manuscripts.
